# Supplementary material for: Unveiling the Bioactive Potential of the Invasive Jellyfish Phyllorhiza punctata Through Integrative Transcriptomic and Proteomic Analyses
Source: Biomolecules. 2025 Aug 4;15(8):1121. doi: 10.3390/biom15081121 (PMC12383608; doi:10.3390/biom15081121)
Supplement: Supplementary file 1 [file biomolecules-15-01121-s001.zip › Suplementary Material/FileS3/PEPFOLD4/XP_065057120_11942/clusters.html]

 PEP-FOLD clusters


Download archive of all models  here   

|  |  |  |
| --- | --- | --- |
| # | file | sOPEP |
| # cluster 1 - Size: 9 |  |  |
|  | XP\_065057120\_11942-model1 | -23.7639 |
|  | XP\_065057120\_11942-model1.1 | -21.2586 |
|  | XP\_065057120\_11942-model1.2 | -19.9444 |
|  | XP\_065057120\_11942-model1.3 | -19.6944 |
|  | XP\_065057120\_11942-model1.4 | -19.0454 |
|  | XP\_065057120\_11942-model1.5 | -19.0363 |
|  | XP\_065057120\_11942-model1.6 | -18.7305 |
|  | XP\_065057120\_11942-model1.7 | -18.5581 |
|  | XP\_065057120\_11942-model1.8 | -18.4071 |
| # cluster 2 - Size: 2 |  |  |
|  | XP\_065057120\_11942-model2 | -21.9592 |
|  | XP\_065057120\_11942-model2.1 | -21.4273 |
| # cluster 3 - Size: 23 |  |  |
|  | XP\_065057120\_11942-model3 | -21.6258 |
|  | XP\_065057120\_11942-model3.1 | -20.7773 |
|  | XP\_065057120\_11942-model3.2 | -20.6875 |
|  | XP\_065057120\_11942-model3.3 | -20.4377 |
|  | XP\_065057120\_11942-model3.4 | -20.3945 |
|  | XP\_065057120\_11942-model3.5 | -20.1101 |
|  | XP\_065057120\_11942-model3.6 | -20.0699 |
|  | XP\_065057120\_11942-model3.7 | -19.7095 |
|  | XP\_065057120\_11942-model3.8 | -19.6809 |
|  | XP\_065057120\_11942-model3.9 | -19.4308 |
|  | XP\_065057120\_11942-model3.10 | -19.4095 |
|  | XP\_065057120\_11942-model3.11 | -19.4048 |
|  | XP\_065057120\_11942-model3.12 | -19.3886 |
|  | XP\_065057120\_11942-model3.13 | -19.3155 |
|  | XP\_065057120\_11942-model3.14 | -19.274 |
|  | XP\_065057120\_11942-model3.15 | -18.9118 |
|  | XP\_065057120\_11942-model3.16 | -18.79 |
|  | XP\_065057120\_11942-model3.17 | -18.7622 |
|  | XP\_065057120\_11942-model3.18 | -18.729 |
|  | XP\_065057120\_11942-model3.19 | -18.2893 |
|  | XP\_065057120\_11942-model3.20 | -18.2081 |
|  | XP\_065057120\_11942-model3.21 | -18.1877 |
|  | XP\_065057120\_11942-model3.22 | -17.5783 |
| # cluster 4 - Size: 7 |  |  |
|  | XP\_065057120\_11942-model4 | -21.5276 |
|  | XP\_065057120\_11942-model4.1 | -19.9965 |
|  | XP\_065057120\_11942-model4.2 | -19.7132 |
|  | XP\_065057120\_11942-model4.3 | -19.5259 |
|  | XP\_065057120\_11942-model4.4 | -18.701 |
|  | XP\_065057120\_11942-model4.5 | -18.5053 |
|  | XP\_065057120\_11942-model4.6 | -17.8563 |
| # cluster 5 - Size: 1 |  |  |
|  | XP\_065057120\_11942-model5 | -21.2498 |
| # cluster 6 - Size: 4 |  |  |
|  | XP\_065057120\_11942-model6 | -21.2303 |
|  | XP\_065057120\_11942-model6.1 | -20.987 |
|  | XP\_065057120\_11942-model6.2 | -18.8295 |
|  | XP\_065057120\_11942-model6.3 | -18.1358 |
| # cluster 7 - Size: 4 |  |  |
|  | XP\_065057120\_11942-model7 | -20.9695 |
|  | XP\_065057120\_11942-model7.1 | -18.4096 |
|  | XP\_065057120\_11942-model7.2 | -18.3936 |
|  | XP\_065057120\_11942-model7.3 | -18.0712 |
| # cluster 8 - Size: 5 |  |  |
|  | XP\_065057120\_11942-model8 | -20.6956 |
|  | XP\_065057120\_11942-model8.1 | -19.048 |
|  | XP\_065057120\_11942-model8.2 | -18.8033 |
|  | XP\_065057120\_11942-model8.3 | -18.6779 |
|  | XP\_065057120\_11942-model8.4 | -18.5317 |
| # cluster 9 - Size: 13 |  |  |
|  | XP\_065057120\_11942-model9 | -20.3782 |
|  | XP\_065057120\_11942-model9.1 | -20.2948 |
|  | XP\_065057120\_11942-model9.2 | -20.1137 |
|  | XP\_065057120\_11942-model9.3 | -19.6845 |
|  | XP\_065057120\_11942-model9.4 | -19.5962 |
|  | XP\_065057120\_11942-model9.5 | -19.4793 |
|  | XP\_065057120\_11942-model9.6 | -19.4315 |
|  | XP\_065057120\_11942-model9.7 | -19.3789 |
|  | XP\_065057120\_11942-model9.8 | -19.3226 |
|  | XP\_065057120\_11942-model9.9 | -18.9167 |
|  | XP\_065057120\_11942-model9.10 | -18.56 |
|  | XP\_065057120\_11942-model9.11 | -18.3751 |
|  | XP\_065057120\_11942-model9.12 | -18.2348 |
| # cluster 10 - Size: 7 |  |  |
|  | XP\_065057120\_11942-model10 | -20.0512 |
|  | XP\_065057120\_11942-model10.1 | -19.5693 |
|  | XP\_065057120\_11942-model10.2 | -19.1355 |
|  | XP\_065057120\_11942-model10.3 | -19.0991 |
|  | XP\_065057120\_11942-model10.4 | -18.8983 |
|  | XP\_065057120\_11942-model10.5 | -18.3722 |
|  | XP\_065057120\_11942-model10.6 | -18.2147 |
| # cluster 11 - Size: 1 |  |  |
|  | XP\_065057120\_11942-model11 | -19.9874 |
| # cluster 12 - Size: 1 |  |  |
|  | XP\_065057120\_11942-model12 | -19.756 |
| # cluster 13 - Size: 1 |  |  |
|  | XP\_065057120\_11942-model13 | -19.4506 |
| # cluster 14 - Size: 1 |  |  |
|  | XP\_065057120\_11942-model14 | -19.3451 |
| # cluster 15 - Size: 3 |  |  |
|  | XP\_065057120\_11942-model15 | -18.7315 |
|  | XP\_065057120\_11942-model15.1 | -18.3307 |
|  | XP\_065057120\_11942-model15.2 | -17.7708 |
| # cluster 16 - Size: 8 |  |  |
|  | XP\_065057120\_11942-model16 | -18.7064 |
|  | XP\_065057120\_11942-model16.1 | -18.5703 |
|  | XP\_065057120\_11942-model16.2 | -18.0937 |
|  | XP\_065057120\_11942-model16.3 | -17.781 |
|  | XP\_065057120\_11942-model16.4 | -17.5738 |
|  | XP\_065057120\_11942-model16.5 | -17.5699 |
|  | XP\_065057120\_11942-model16.6 | -17.4638 |
|  | XP\_065057120\_11942-model16.7 | -17.3617 |
| # cluster 17 - Size: 3 |  |  |
|  | XP\_065057120\_11942-model17 | -18.6971 |
|  | XP\_065057120\_11942-model17.1 | -18.0907 |
|  | XP\_065057120\_11942-model17.2 | -17.713 |
| # cluster 18 - Size: 2 |  |  |
|  | XP\_065057120\_11942-model18 | -18.5887 |
|  | XP\_065057120\_11942-model18.1 | -18.1079 |
| # cluster 19 - Size: 2 |  |  |
|  | XP\_065057120\_11942-model19 | -18.4106 |
|  | XP\_065057120\_11942-model19.1 | -17.9093 |
| # cluster 20 - Size: 1 |  |  |
|  | XP\_065057120\_11942-model20 | -18.3582 |
| # cluster 21 - Size: 1 |  |  |
|  | XP\_065057120\_11942-model21 | -18.3334 |
| # cluster 22 - Size: 1 |  |  |
|  | XP\_065057120\_11942-model22 | -17.8331 |
|  |  |  |
